# Design of an electromagnetic metallic metamaterial absorber for terahertz applications

**DOI:** 10.1038/s41598-023-47825-y

**Published:** 2023-12-12

**Authors:** Ahmed S. Elkorany, Fatma S. Saeed, Ahmed A. Hassan, Demyana Adel A. Saleeb

**Affiliations:** 1 Department of Electronics and Electrical Communication Engineering, Faculty of Electronic Engineering, Menoufia University, Menouf, 32952 Menoufia Egypt; 2 Faculty of Engineering, Kafrelsheikh University, Kafrelsheikh, 33516 Egypt

**Keywords:** Engineering, Electrical and electronic engineering

## Abstract

Metamaterial absorbers have diverse applications in the terahertz range. In this paper, a metallic metamaterial absorber is designed to work as a narrowband, wideband, or ultrawideband according to application. A systematic method with minimal computational requirements for the design is developed. The method is efficient since the only information required is the operating frequency and required bandwidth. A condition for zero reflection which ensures matching with free space is derived. The proposed design method is used for the design of narrowband, wideband, and ultrawideband absorbers. For each case the design parameters are different. The narrow bandwidth is less than 5%, while the wide band lies between 5 and 20%, and the ultrawideband is larger than 20%. The dimensions of the designed structure and material properties (ε_r_″) are different for each case. The designed absorber is wide-angle and polarization-independent. The sensitivity of the designed material due to changes of physical dimensions and permittivity is studied. To validate results, reflectivity and absorptivity are calculated using CST package. The dimensions obtained from the developed method are a bit modified using trial and error. The results from the developed method and CST are in excellent agreement.

## Introduction

Electromagnetic metamaterial absorbers are very useful to construct active and passive components for imaging from microwave frequencies to visible wavelengths^1–3^. Metamaterial absorbers can be classified into three types^4^: metallic absorbers, all-dielectric absorbers, and coherent absorbers. At terahertz frequencies, it is difficult to find naturally occurring materials with strong absorption coefficients that are also compatible with standard microfabrication techniques.

In^5^, a terahertz biosensor based on all-metal metamaterial is proposed. The biosensor uses stainless steel materials manufactured using laser-drilling technology. The unit cell is a hollow-dumb pattern. A temperature-tuneable terahertz perfect absorber composed of a periodic micro-cross-shaped structure of the strontium (STO) titanate resonator is proposed in^6^. The perfect absorption can also be achieved by using a 3-D square, circular, or ring STO structure. The structure is complicated. A broadband visible perfect absorber using a plasmonic metasurface which consists of a quadrilateral truncated cone configuration is designed in^7^. The unit cell consists of 4 layers: GaAs, Ti, SiO_2_, and Cu with complicated geometry. A broadband metamaterial absorber is presented in^8^. The unit cell is of complicated geometry with loaded four surface-mounted resistors.

Theoretical approaches and algorithms such as impedance matching theory^9^, reflection theory^10^, antenna reciprocity theory^11^, and optimization algorithms^12,13^, were introduced to analyze broadband absorbers. However, all these theories cannot guide the design of wideband absorbers. They only explain the broadband absorbing mechanism.

In this paper, a metallic metamaterial absorber was designed to operate in the terahertz (1–3 THz) range. The design depends upon the scale-invariant property of Maxwell’s equations with no charges or currents.

Scale invariance is a term used in mathematics, economics, and physics and is a feature of an object that does not change if all scales in the object are multiplied by a constant factor^14,15^. Maxwell’s equations with no charges or currents are an example of scale invariance. If E(x,t) is a solution of Maxwell’s equations, then E(λx, λt) is also a solution, where λ is a constant.

The novelty in the paper lies in the fact that narrowband absorbers, wideband absorbers, and ultrawideband absorbers can be designed using the same developed design technique. The dimensions and dielectric properties of the designed absorber depend on the bandwidth. The developed design technique is: (1) based on simple mathematical equations, (2) efficient, since the only required information is the operating frequency and bandwidth, and (3) does not require expensive software packages. Just a simple MATLAB program is required. The designed absorber is wide-angle and polarization-independent. Finite integration technique (CST) was used to design narrowband, wideband, and ultrawideband absorbers. There was little difference between the results obtained and those obtained by the developed technique. However, the dimensions obtained by the developed technique were slightly modified and the results obtained were in excellent agreement with those obtained by the finite integral technique.

A microwave metal metamaterial absorber (6–14 GHz) was designed by the authors^16^. This absorber is scaled up in frequency to operate in the THz band (1–3 THz).

The paper is organized as follows. Section “Theory” includes theory and derivation of condition for zero reflection. A simple procedure for the design of the absorber is developed in section “Design”. Results and Conclusions are given in subsequent sections.

## Theory

**Figure 1:**
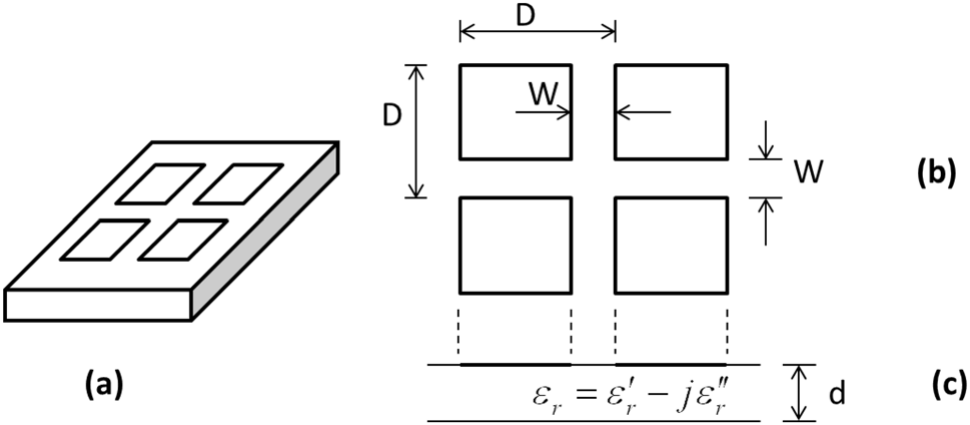
The thin absorber (**a**), top view (**b**), side view (**c**).

The absorbing structure is shown in Fig. 1. The dimensions of the structure must be small compared to wavelength.

The input impedance to such structure is given by^17^:

1 $$Z_{inp}^{TE} = \frac{{j\omega \mu_{o} d}}{{1 - 2k_{eff} \propto d\left( {1 - \frac{{sin^{2} \theta }}{{\varepsilon_{r} + 1}}} \right)}}$$

2 $$Z_{inp}^{TM} = \frac{{j\omega \mu_{o} d\left( {1 - \frac{{sin^{2} \theta }}{{\varepsilon_{r} }}} \right)}}{{1 - 2k_{eff} \propto d\left( {1 - \frac{{sin^{2} \theta }}{{\varepsilon_{r} }}} \right)}}$$

3 $$k_{eff} = k_{o} \sqrt {\epsilon_{eff} }$$

4 $$\in_{eff} = \frac{{ \in_{r} + 1}}{2}$$

5 $$\propto = \frac{{k_{eff} D}}{\pi }\ln \left( {\frac{2D}{{\pi w}}} \right)$$

In deriving Eqs. (1) and (2) it was assumed that w/D  ≪ 1. The substrate is a lossy dielectric material. Therefore, the dielectric constant is complex:

6 $$\epsilon_{r} = \epsilon_{r}{\prime} - j\epsilon_{r}^{^{\prime\prime}}$$

**Figure 2:**
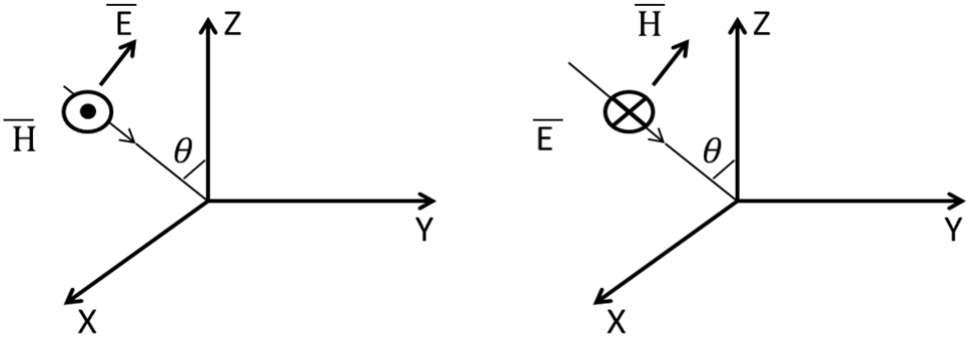
The incident wave.

θ is the angle of incidence as shown in Fig. 2.

If the dielectric constant ε_r_ is chosen to be large, the input impedance of Eqs. (1) and (2) will be independent of θ. This means that the absorbing material is independent of polarization. The input impedance becomes:

7 $$Z_{inp}^{TE} = Z_{inp}^{TM} = \frac{{j\omega \mu_{o} d}}{{1 - 2k_{eff} \propto d}}$$

This equation can be put in the form:

8 $$Z_{inp} = \frac{j\omega L}{{1 - \omega^{2} LC^{\prime}}}$$

where

9 $$L = \mu_{o} d$$

10 $$C^{\prime} = \epsilon_{o} (\varepsilon_{r} + 1)\frac{D}{\pi }\ln \left( {\frac{2D}{{\pi w}}} \right)$$

If ε_r_ in Eq. (10) is replaced by its value in (6), after some manipulation we have:

11 $$C^{\prime} = \epsilon_{o} \left( {\epsilon_{r}{\prime} + 1} \right)\frac{D}{\pi }\ln \left( {\frac{2D}{{\pi w}}} \right) - j\epsilon_{o} \epsilon_{r}^{^{\prime\prime}} \frac{D}{\pi }\ln \left( {\frac{2D}{{\pi w}}} \right)$$

Equation (11) can be put in the form:

12 $$C^{\prime} = C - jg \ldots \; \ldots \; \ldots .$$

where:

13 $$g = \epsilon_{o} \epsilon_{r}^{^{\prime\prime}} \frac{D}{\pi }\ln \left( {\frac{2D}{{\pi w}}} \right)$$

and:

14 $$C = \epsilon_{o} \left( {\epsilon_{r}{\prime} + 1} \right)\frac{D}{\pi }\ln \left( {\frac{2D}{{\pi w}}} \right)$$

From (12) into (8) we get (omitting details):

15 $$\begin{aligned} Z_{{{\text{inp}}}} & = \, \omega^{3} {\text{L}}^{2} {\text{g / }}\left[ { \, ( \, 1 - \, \omega^{2} {\text{L C }})^{2} + \, ( \, \omega^{2} {\text{L g }})^{2} } \right] \\ & \quad + {\text{ j }}\omega {\text{ L }}( \, 1 \, - \, \omega^{2} {\text{L C }}) \, / [ \, ( \, 1 - \, \omega^{2} {\text{L C }})^{2} + \, ( \, \omega^{2} {\text{L g }})^{2} ] \\ \end{aligned}$$

reflection coefficient of the structure is given by:

16 $$R = \frac{{Z_{inp} - \eta }}{{Z_{inp} + \eta }}$$

where η is the intrinsic impedance of free space.

For the structure to work as a perfect absorbing material, there must be matching with free space. This means R must be zero which means Z_inp_ = η (real). The imaginary part of (15) must be zero. Thus:

17 $$\omega = \frac{1}{{\sqrt {LC} }}$$

At this frequency, the input impedance from (15) becomes:

18 $$Z_{{{\text{inp}}}} = \, \eta \, = \, 1/\omega {\text{g}}$$

The impedance given by (18) is the maximum value of input impedance. The bandwidth of the input impedance is obtained from:

19 $$Z_{{{\text{inp}}}} = \frac{\eta }{\sqrt 2 }$$

From which the bandwidth is given by :

20 $$B = \frac{1}{\eta }\sqrt{\frac{L}{C}}$$

### Ethics approval

The authors assure that this paper is the authors’ own original work, which has not been previously published elsewhere. The paper is not currently being considered for publication elsewhere. The paper reflects the authors’ own research and analysis in a truthful and complete manner.

### Consent to participate

All authors agreed to participate in this research.

## Design

From the theory presented above, it is possible to develop a simple procedure to design (find the dimensions of) the absorbing material. The data available is the operating frequency and bandwidth. From Eqs. (17), (18) and (20) we have:

21 $$g = \frac{1}{\omega \eta }$$

22 $$L = \frac{\eta B}{{2\pi f}}$$

23 $$C = \frac{1}{2\pi f\eta B}$$

Using the values of g, L, and C together with Eqs. (9), (13), and (14) we get the dimensions of the structure d, D, w, ε_r_′, and ε_r_″. But we need some simplifying assumptions. It was mentioned that w/D must be small. We take w/D to be 0.1. Take ε_r_′ to be large (= 10). Thus we are left with 3 unknowns: d, D, and ε_r_″ with 3 Eqs. (9), (13) and (14). Thus all the dimensions can be found.

## Results

**Figure 3:**
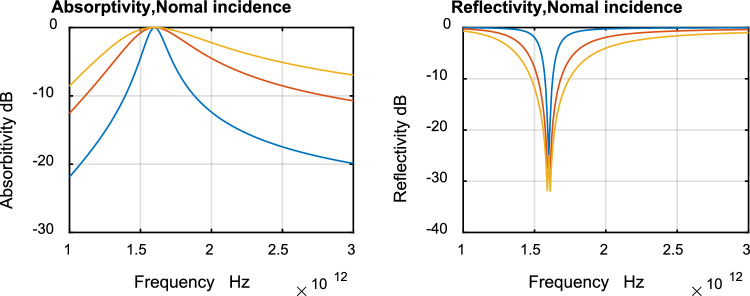
Absorptivity (**a**) and reflectivity (**b**) for normal incidence, narrowband (blue), wideband (red), ultrawideband (yellow).

Narrowband absorbers are required for spectrally selective absorbers which has potential applications in sensitive detectors and narrowband thermophotovoltaic emitters^18^. Broadband absorbers are required for energy harvesting: solar-driven steam generation and photodetection via hot electron harvesting^19^. Here we show results for narrowband and broadband absorbers. Figure 3 shows absorptivity vs. frequency (a), and reflectivity vs. frequency (b) for normal incidence. Table 1 summarizes the properties of different absorbers.

**Table 1:** Summary of properties of different absorbers.

	Frequency (THz)	Bandwidth	Permittivity (real)	Permittivity (imaginary)	Periodicity (D) (µm)	Distance between patches (W) (µm)	Substrate thickness (d) (µm)
Narrow band	1.6	0.05	10	0.55	92	9.2	1.5
Narrow band_cst	1.6	0.05	10	0.55	95	9.5	1.05
Wide band	1.6	0.15	10	1.65	30.7	3.07	4.47
Wide band_cst	1.6	0.15	10	1.65	25	2.5	4
Ultra wide band	1.6	0.25	10	2.75	18.4	1.84	7.46
Ultrawide band_cst	1.6	0.25	10	2.75	17.5	1.75	6

**Figure 4:**
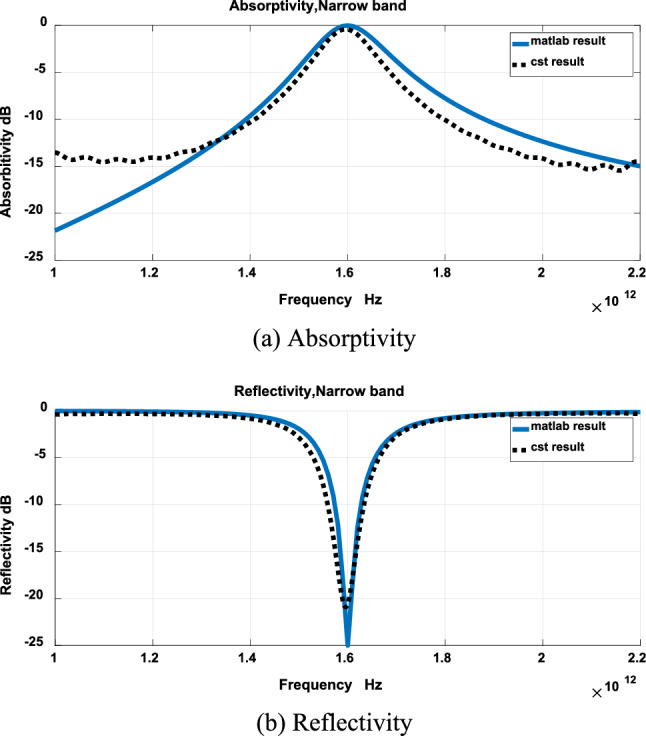
Narrow band.

**Figure 5:**
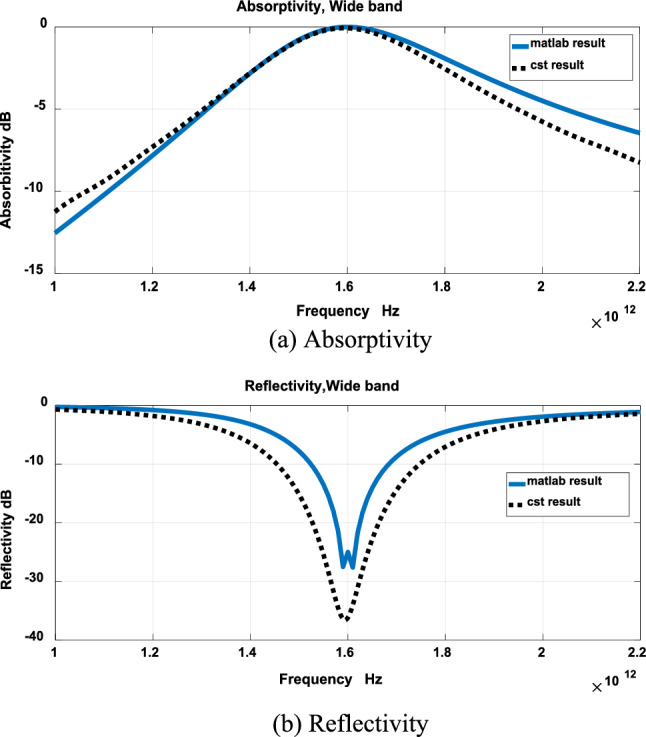
Wide band.

**Figure 6:**
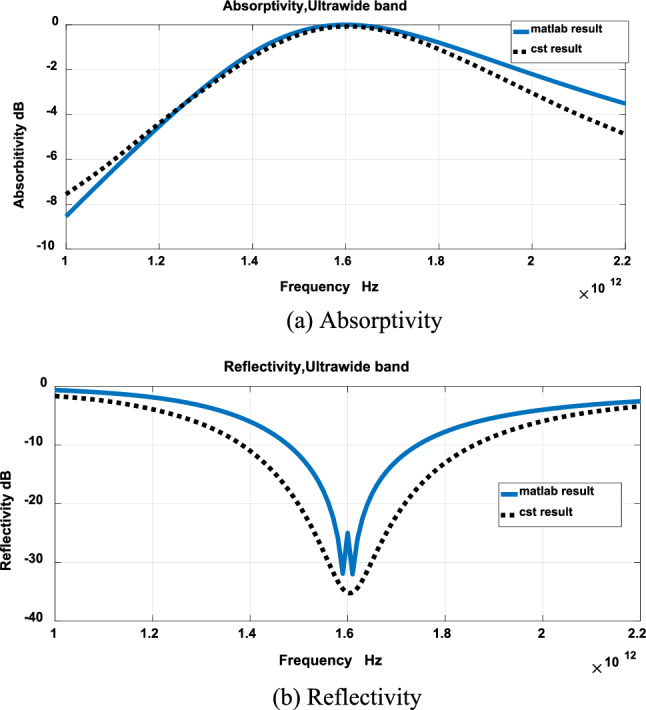
Ultrawide band.

The results of Fig. 3 were validated using CST. Figures 4, 5 and 6 show comparison between results obtained by the developed technique and CST for narrow band, wideband and ultrawide band respectively. The dimensions obtained by the developed technique were modified to get these results.

**Figure 7:**
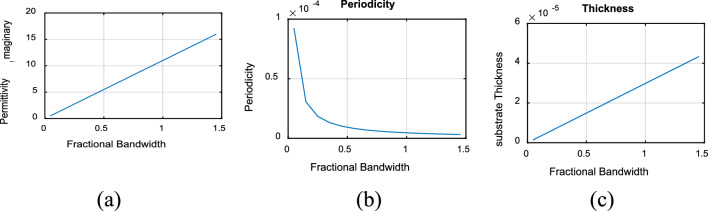
Variation of (**a**) imaginary part of permittivity, (**b**) periodicity, and (**c**) substrate thickness with fractional bandwidth.

The maximum bandwidth that can be realized depends upon the dimensions of the structure and the material available. The choice of dimensions depends upon technology of fabrication. Figure 7 shows variation of imaginary part of permittivity, periodicity, and substrate thickness with fractional bandwidth. Increase of fractional bandwidth means increase of substrate thickness and imaginary part of permittivity while periodicity is reduced.

**Figure 8:**
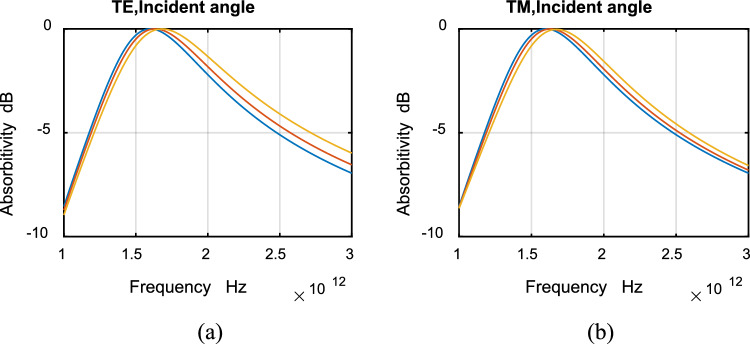
Absorptivity with frequency for ultrawideband TE and TM polarizations. Incident angle: 0° (blue), 40° (red), 80° (yellow).

**Figure 9:**
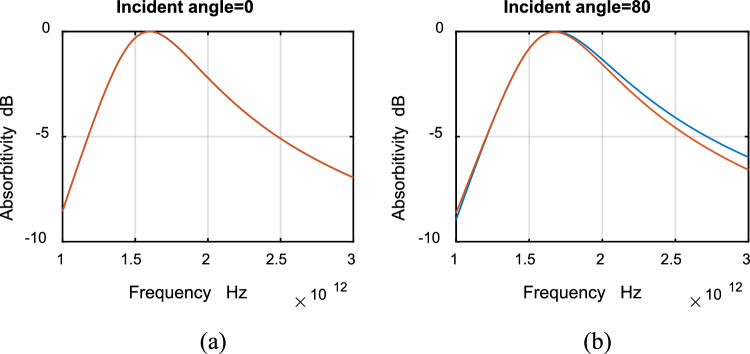
Absorptivity with frequency for ultrawideband TE (red) and TM (blue) polarizations. Incident angle: (**a**) 0°, (**b**) 80°.

The absorptivity for TE and TM polarizations at different angles of incidence are shown in Fig. 8a,b for ultrawideband absorbers. Figure 9 shows absorptivity for TE and TM ultrawideband absorbers for incident angle (a) 0°, (b) 80°. It can be noticed from the figures that the difference in absorptivity between TE and TM modes is extremely small. Thus the designed absorbers are polarization independent. As the incident angle increases, no change in absorptivity can be noticed except for a very small shift in resonance frequency. The designed absorber is wide angle.

**Figure 10:**
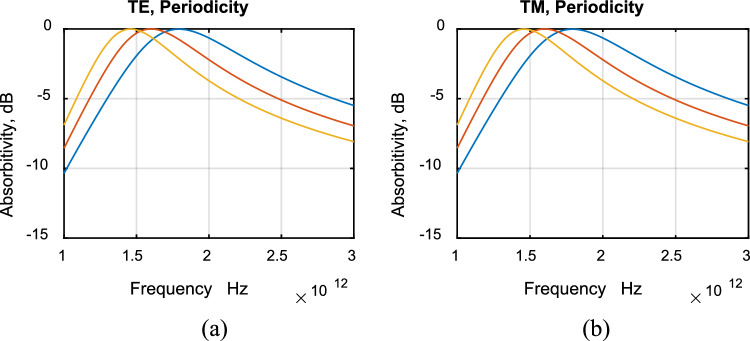
Sensitivity to change of periodicity.

**Figure 11:**
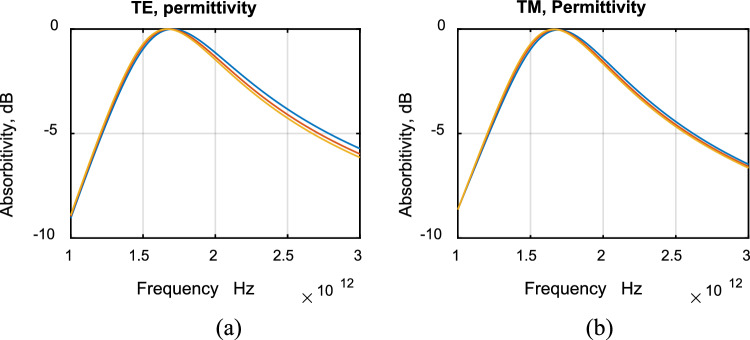
Sensitivity to change of permittivity.

The sensitivity of absorptivity to changes of periodicity (D) and permittivity (ε_r_′) was studied. Figure 10 shows changes of absorptivity due to a 20% change of periodicity. This caused a 12.5% change of resonance frequency. Figure 11 shows changes of absorptivity due to a change of the real part of relative permittivity (ε_r_′) from 8 to 12. The change of absorptivity is very small and can hardly be noticed.

The power imparted per cubic meter of dielectric substrate material from the RF field is: P = 2π ε_o_ ε_r_″ f E^2^ where f is the frequency and E is the electric field. The work suggested in this study is superior to other published studies in terms of wide angle, simple structure, and simple mathematics used. This is indicated in Table (2).

**Table 2:** Comparison with other works.

References	Materials	Operating frequency	Geometry of unit cell	Computation	Incident angle
Gangqi Wang (2021)^5^	All-metal Stainless steel	0.48 THz	Hollow dumb pattern	CST	NA
Jingcheng Zhao (2022)^6^	Strontium titanate STO	0.1–0.3 THz	Cross shaped, or 3D square, circular or ring	HFSS	NA
Yicheng Wang^7^ (2022)	GaAs, Ti, SiO2, Cu	490–772 nm (wavelength)	4 layers, truncated cone	FDTD	0°–60°
Ahmed S. Saadeldeen (2023)^8^	Top metallic layer	14.35–29.18 GHz	Complicated metallic pattern	HFSS	0°–50°
This work	Metallic patch	1.6 THz	Just planar metallic patch	Simple MATLAB programs	0°–80°

## Conclusions

A perfect absorber with absorptivity 100% has been designed at 1.6 THz. The scale invariance property of Maxwell’s equations with no charges or currents was used to scale up a design from microwave to terahertz range. A simple procedure was developed for the design. The results were validated using CST with little modification of the dimensions obtained by the developed procedure. The bandwidth of the absorber can be adjusted through physical parameters of the absorber. The physical parameters of the absorber were obtained for bandwidths 5, 15, and 25%. The results obtained showed that the absorber is wide angle and polarization independent. A 20% change of periodicity (D) caused a 12.5% change of resonance frequency. The change in absorptivity due to change of (ε_r_′) from 8 to 12 was too small to be noticed.

## Data Availability

The authors affirm that the data underlying the study's conclusions are presented in the article, and the manuscript has no associated data. All data generated or analysed during this study are included in this published article.
